# Supplementary figures and images for: Comparative and evolutionary analysis of the reptilian hedgehog gene family (Shh, Dhh, and Ihh)
Source: PeerJ. 2019 Aug 30;7:e7613. doi: 10.7717/peerj.7613 (PMC6718155; doi:10.7717/peerj.7613)

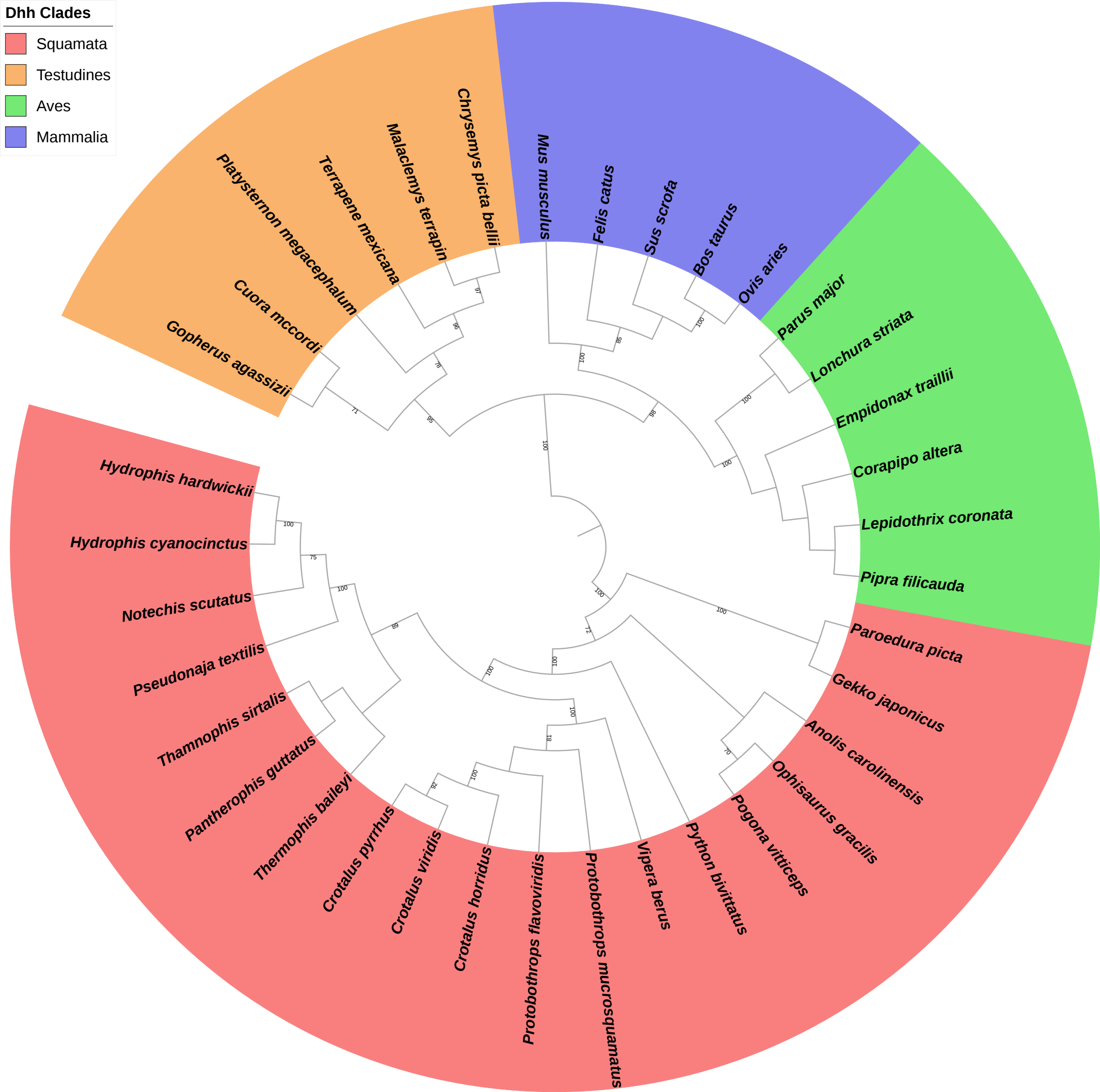

Supplement: Supplemental Information 1 [file peerj-07-7613-s001.pdf]

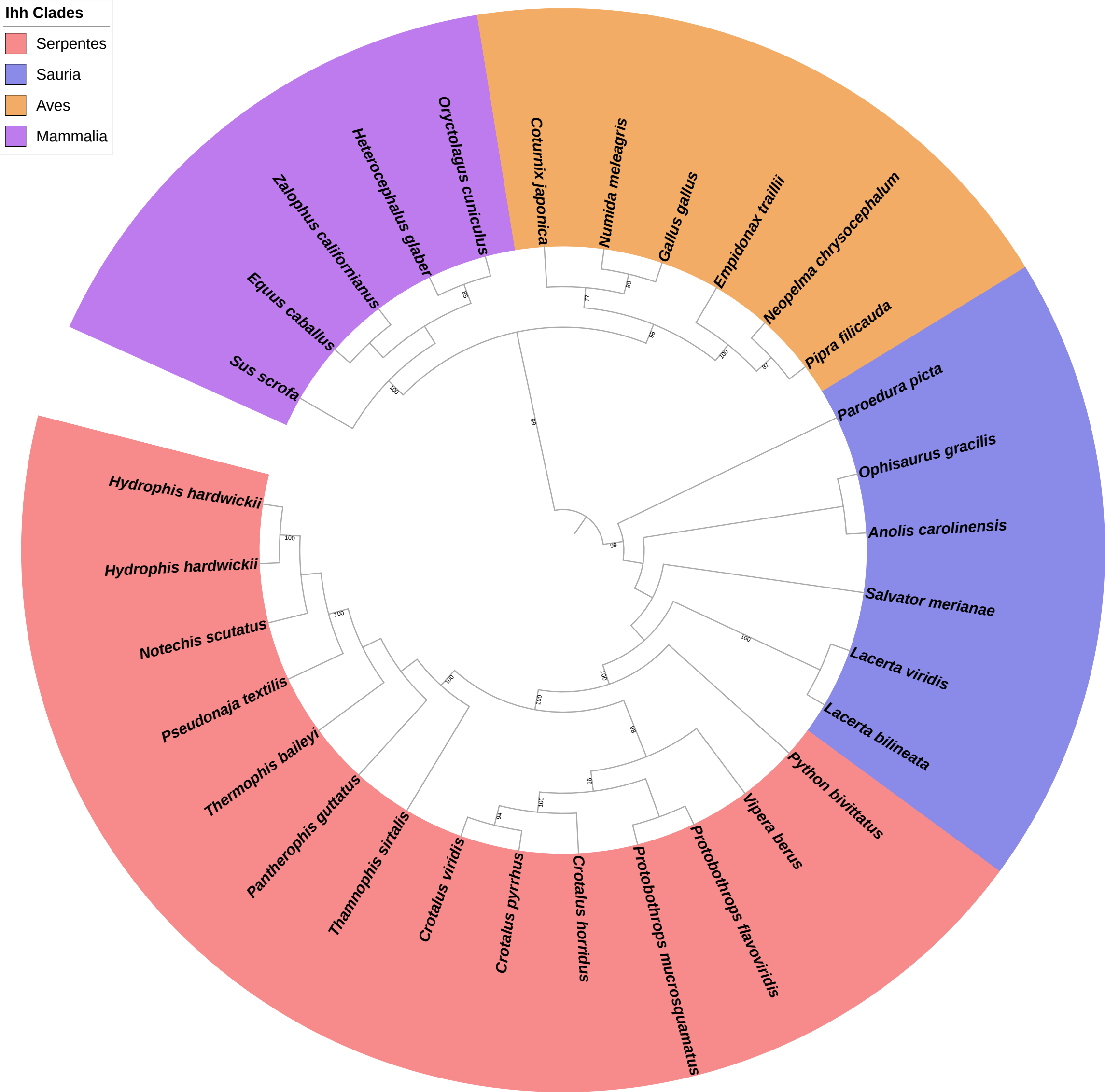

Supplement: Supplemental Information 2 [file peerj-07-7613-s002.pdf]
