# Supplementary material for: Comparative and evolutionary analysis of the reptilian hedgehog gene family (Shh, Dhh, and Ihh)
Source: PeerJ. 2019 Aug 30;7:e7613. doi: 10.7717/peerj.7613 (PMC6718155; doi:10.7717/peerj.7613)
Supplement: Supplemental Information 3 [file peerj-07-7613-s003.docx]

| Species  Scientific Name | Common name | Genome Assembly Number | Contig N50  (kbp) | Depth  (×) |
| --- | --- | --- | --- | --- |
| *Crocodylus porosus* | Australian saltwater crocodile | GCA_001723895.1 | 34.1 | 74 |
| *Malaclemys terrapin* | Diamondback terrapin | GCA_001728815.2 | 437.2 | 16 |
| *Cuora mccordi* | McCord's box turtle | GCA_003846335.1 | 74.3 | 71 |
| *Chelonoidis abingdonii* | Abingdon island giant tortoise | GCA_003597395.1 | 73.2 | 28 |
| *Gopherus agassizii* | Agassiz's desert tortoise | GCA_002896415.1 | 43.7 | 118 |
| *Platysternon megacephalum* | Big-headed turtle | GCA_003942145.1 | 213.6 | 208 |
| *Crotalus viridis* | Western rattlesnake | GCA_003400415.1 | 15.7 | 100 |
| *Vipera berus* | Adder | GCA_000800605.1 | 11.7 | 121 |
| *Salvator merianae* | Argentine black and white tegu | GCA_003586115.2 | 500.0 | 134 |
| *Lacerta viridis* | Green lizard | GCA_900245905.1 | 662.5 | 48 |
| *Protobothrops flavoviridis* | Habu | GCA_003402635.1 | 18.9 | 96 |
| *Lacerta bilineata* | Western green lizard | GCA_900245895.1 | 368.2 | 48 |
| *Paroedura picta* | Panther gecko | GCA_003118565.1 | 15.2 | 75 |
| *Pantherophis guttatus* | Corn snake | GCA_001185365.1 | 2.4 | 13 |
| *Ophiophagus hannah* | King cobra | GCA_000516915.1 | 5.2 | 28 |
| *Crotalus pyrrhus* | White Speckled Rattlesnake | GCA_000737285.1 | 5.3 | 40 |
| *Hydrophis cyanocinctus* | Asian annulated sea snake | GCA_004023725.1 | 7.4 | 100 |
| *Hydrophis hardwickii* | Hardwick's sea snake | GCA_004023765.1 | 5.4 | 86 |
| *Thermophis baileyi* | Bailey's Snake | GCA_003457575.1 | 2414.0 | 185 |
| *Ophisaurus gracilis* | Anguidae lizard | [SRP052050](http://www.ncbi.nlm.nih.gov/sra?term=SRP052050) | 1.3 | 86 |
