# Supplementary material for: Comparative and evolutionary analysis of the reptilian hedgehog gene family (Shh, Dhh, and Ihh)
Source: PeerJ. 2019 Aug 30;7:e7613. doi: 10.7717/peerj.7613 (PMC6718155; doi:10.7717/peerj.7613)
Supplement: Supplemental Information 6 [file peerj-07-7613-s006.docx]

| Gene | No. of species | LnL M7 | LnL M8a | LnL M8 | LRT P-value(M8a vs M7) | LRT P-value(M8 vs M7) | M8 | SLAC | FEL | REL | Total no.of sites |
| --- | --- | --- | --- | --- | --- | --- | --- | --- | --- | --- | --- |
|  |  |  |  |  |  |  |  |  |  |  |  |
| Shh | 44 | -11291.7131 | -11287.93888 | -11288.28972 | 0.006006005 | 0.0326014 | 0 | 0 | 0 | 0 | 0 |
| Dhh | 36 | -9739.78488 | -9738.99414 | -9739.788706 | 0.208544763 | 0.9961574 | 0 | 1 | 0 | 6 | 0 |
| Ihh | 31 | -8249.68907 | -8250.071524 | -8249.689141 | 0.381799417 | 0.99995 | 0 | 0 | 0 | 0 | 0 |
